# Electricity and Its Use in Medicine. II

**Published:** 1894-04-14

**Authors:** S. W. Wheaton

**Affiliations:** Physician to the Royal Hospital for Children and Women


### Medical Progress and Hospital Clinics.

[*The Editor will be glad to receive offers of co-operation and contributions from members of the profession. All letters should be addressed to* The Editor, The Lodge, Porchester Square, London, W.]

### ELECTRICITY AND ITS USE IN MEDICINE.—II.

By S. W. Wheaton, M.D., M.R.C.P., D.P.H., Physician to the Royal Hospital for Children and Women.

Now, with respect to the use of electricity in medicine. All that is required is a battery provided with a coil for producing the interrupted current; a battery for supplying constant currents; some insulated wires; metal electrodes covered with lint, which can be soaked in salt and water for application of the current; and a galvanometer graduated so as to show the strength of the current which is actually passing through the patient. The strength of the current which is passing through the body is very much less than the strength of the current when it does not pass through the body, and this diminution is due to the resistance which the skin and other tissues of the patient oppose to the current, and by which part of the energy of the current is converted into heat. The current is also lessened by an opposing electromotive force which is set up in the body from electrolysis. Owing to these facts, the strength of the current when the circuit is outside the body is no guide to its strength when passing through the body; hence the necessity of measurement by means of a galvanometer in the circuit, and the numerous fallacies which are found in text-books with regard to this point. Owing to the heat developed in the skin large burns have been frequently produced by careless operators. To avoid this the current should be diffused over as large an area as possible, if it is employed in the continuous form. The electrodes for application of the constant current should be large, their surface should be perfectly smooth. If one point is nearer the skin than another the greater part of the current will run through this point, and very often produce a punched-out burn on the skin; hence the electrodes should be applied with even pressure all over them. Always use the interrupted current in preference to the continuous one, if either will serve your purpose, especially if you are a beginner, for you can do no harm with it if you remember never to employ it under the following circumstances. (1) Never to employ it in the case of muscles which have only recently become paralysed, but always give them time to rest for three or four weeks before applying the current; (2) never to use it when you suspect any acute mischief in brain or cord; (3) never to use it over the cardiac region or along the course of the vagus nerve, except it is required to stimulate the heart in cases of threatening death from asphyxia or anæsthetics.

The differences between the two kinds of current are, in the first place; that the interrupted current, owing to its short duration, has not time to produce the heat, electrolysis, or osmosis, which are produced by the constant current, to any appreciable extent; secondly, it acts more vigorously as a stimulus of healthy muscle, whereas the continuous current alone will not produce contraction, except when interrupted by making and breaking the contact.

I must caution you to be most careful in the use of the constant current; you should always have a galvanometer in the circuit, so that you can tell what amount of current is actually passing through the patient, and, as a rule, the current passing through the body, or part of the body, should not exceed 30 milliamperes in strength. If you are not careful to watch the strength of your current, and to see that your electrodes are properly applied, you may easily burn a hole in your patient's skin, if nothing worse happens. I will now give a few examples of the use of electricity in disease, and will remind you that its action must be due to either heat, electrolysis, osmosis, or to its actions on vessels, muscles, or nerves, which have been before described. I shall say nothing of those mysterious properties which are attributed to it by the quack and impostor, and which are believed in by their victims.

The first case that I will bring under your notice is that of a young girl who has suffered from incontinence of urine both at day and night time since childhood. Her age is eighteen years, she is otherwise healthy, and has been treated with many drugs by many medical men. I tried large doses of belladonna without any effect. I then applied the faradic current, using a metallic bladder sound as an electrode, to the handle of which one pole of the coil was connected, covering it over except an inch of the tip with indiarubber tubing to insulate it, and after inserting the tip into the urethra; the other pole of the coil was connected to a flat metal electrode, and applied to the lumbar-spine. It is noticed that the urethra is dilated and flacid. After one application she is perfectly cured. Now can you explain this? I believe that here the current acts by opening up the disused nerve tracks, and breaking down their resistance, so that the sleeping cells behind them can come into action and the tone of the sphincter is restored. This treatment is equally successful in boys, in whom I use the same electrode, but pass it into the rectum, so that the uninsulated point is pressing against the base of the bladder. I have never seen this treatment fail. Of course you must first see that the incontinence is not due to stone in the bladder, polypus of the rectum, or other gross cause. Case two is a girl aged twenty years, of dark complexion, and nervous aspect, with no other disease except the constant recurrence of attacks of what may be vulgarly but expressively called "belching of wind." These attacks come on many times a day, and end with the bringing up of a little watery fluid. The tongue is clean, and she is well nourished. On examining the abdomen, the stomach area of resonance is found to be enormously increased, quite obliterating part of the cardiac dulness, and passing high up into the thorax.

This is a case of dilatation of the stomach, it is equally common also in young men, and such people are a great nuisance to themselves and to other people; hence they avoid society, and become melancholic and perhaps suicidal. I used here the faradic current over the stomach. One pole is used in the form of a lead plate placed over the lower dorsal spine, and on which the patient lies, the other pole is moved about over the stomach. The result at first is rather alarming, the area of stomach resonance becomes larger, and the patient is seized with an attack of uncontrollable "belching." This action of "belching" is, I believe, caused by spasm of the diaphragm compressing the stomach, which is distended with gas, and squeezing the gas out in an irregular manner. After a few minutes the stomach becomes smaller, and the gas is all expelled. After six applications the patient was cured. I saw her a few days ago, and she remains well and free from the attacks. The result in this case is I think, due to the direct stimulation of the muscle fibre of the stomach, by which it recovers its lost power and no longer allows of distension. The preliminary increase in the area of stomach resonance, which I have seen occur in other cases, I am unable to explain; nor can I explain the mechanism by which in this affection such a large amount of non-fetid gas is poured out into the stomach. Case three is a young woman, aged twenty-two years, who was admitted into the hospital with signs of early phthisis at the apex of the left lung; soon after admission she was seized with attacks of dyspnœa and stridor in breathing. On examination there was found mild laryngeal catarrh and abductor paralysis of the larynx, both cords being close together and almost immovable. The attacks recurred again and again, the dyspnœa was so great that the house surgeon thought that tracheotomy would be required, since the attacks became worse towards night. I instructed him, when the patient had the next attack, to apply the faradic current, with the two poles, one on each side of the larynx; he did so, and to his surprise the attacks ceased and never recurred again. I have seen the patient several times since, and she continues well, with the exception some signs of phthisis, and is free from laryngeal attacks. I have seen several cases exactly similar to this one. There is always in these cases a little catarrh of the larynx, and the patients are neurotic. Temporary loss of conducting power in the laryngeal nerves seems to be caused by the catarrhal inflammation, and the nervous impulses seem to be unable to find their way through them until the way has been reopened by the current. The wonderful effects of electricity in hysteria are of difficult explanation, but I think that, as in the case just mentioned, they are due to the reopening up of the nervous tracts which have become clogged or thickened, so to speak, by disuse. The battery is of the greatest use to the practitioner in cases of hysteria, for you can always use the battery, and the patient's friends will assist you in its application. Case four was one of diabetes, a boy aged eleven years. This boy's father died in St. Thomas's Hospital from diabetes when I was house physician there. The boy was admitted under my care in June, 1891, at the Waterloo Road Hospital; his weight on admission was 4 st. 2 lbs., specific gravity of urine 1,045, and the urine contained a large amount of sugar, which was estimated throughout by Pavy's method. He remained in the hospital until April, 1892, when he was discharged, weighing 4 st. 7 lbs. The specific gravity of the urine was 1,036, and it contained just half the amount of sugar which it contained on admission to hospital. From this it will be seen that the progress of the disease was arrested, and when I last saw him, about a month ago, he continued well, and as far as outside appearances went, looked in perfect health, but there was still a small amount of sugar in the urine. The method which I adopted in this case, after trying almost all the known kinds of treatment for diabetes, including diet, opium, codeia, and sodium salicylate, without any improvement, was the application of a strong, constant current across the liver, from the spine behind in the lower dorsal region, to the epigastrium in front, a current of fifteen milliamperes usually passing through the patient. This was continued every day, for periods of from one to three hours, for weeks at a time, and was then discontinued and resumed several times. The improvement and arrest of the disease in a case of such bad prognosis, owing to the youth of the patient and marked hereditary tendency, I attribute to the constant current thus used. Dr. Pavy has shown that diabetes is probably due to permanent dilatation of the branches of the hepatic artery, by which too much arterial blood is brought to the liver. The current acted in the above case, I think, by passing along the splanchnic nerves to the coats of the branches of the hepatic artery, in this way causing them to contract and become smaller, and thus diminishing the supply of arterial blood to the liver.. Case five was one of intractable neuralgia in the left arm of a lady aged thirty; after continued trial of nearly all remedies, with no improvement, I applied the constant current from the neck to the arm, using lead strips in the form of bandages as an electrode for the arm, and a lead plate over the brachial plexus in the neck. This treatment was followed by a permanent cure after six applications. Case six was one of a young lady with an ulcer over the back of the wrist, following a carbuncle in this position; the ulcer refused to heal with treatment or rest. I applied a small lead plate, cut to the shape of the ulcerated surface, as an electrode, and passed the constant current from the back of the neck to the ulcer. The first application was followed by formation of vesicles around the ulcer and much pain, but after eight applications it has soundly healed. In the two last cases the current has acted, I think, by improving the general nutrition of the parts; this improvement is, I believe, chiefly due to the local development of heat in the line of the current, and the bringing of more blood to the part by dilating the vessels. The great influence of heat in quickening the growth and improving the nutrition and vigour of cells is well known. I could relate to you many more interesting cases, but time will not allow; but you will, perhaps, expect me to say a few words about the use of electricity in diseases of women, especially in fibroid tumours of the uterus. In these cases I believe that the effects are entirely due to heat. Powerful currents are used, and a platinum electrode is placed in the uterus. Now platinum is a comparatively bad conductor, and becomes heated by the current passing through it, and thus cauterises the interior of the uterus. Large flat plates are used for the abdominal electrodes, but even with these electrodes I have often seen small ulcers produced by the heat at the points on the skin where pressure has been greatest. I need hardly speak of the great care which is required in its use, inasmuch as cases have been reported where severe burns on the vaginal wall have been produced owing to the concentration of the current at one spot. Suppose there be any collection of pus in the pelvis in the neighbourhood of the uterus, consider what the effect of such heat will be upon it? I need hardly say that it will cause it to decompose, and the patient may be exposed to great danger. The current, I believe, stops the hæmorrhage and the growth of the tumour by destroying the vessels of the mucous membrane of the uterus by the heat which is produced; and I think that the removal of the mucous membrane by the curette is a safer line of treatment to adopt in most instances, or, at any rate, in those instances in which the uterine canal is not so much narrowed or distorted as to prevent the effectual use of the curette.

